# Social Mobility and Health-Related Quality of Life Trajectory Classes Among Older Women and Men

**DOI:** 10.1177/08982643241242513

**Published:** 2024-04-01

**Authors:** Anna-Maria Lahti, Tuija M. Mikkola, Niko S. Wasenius, Timo Törmäkangas, Jenni N. Ikonen, Sini Siltanen, Johan G. Eriksson, Mikaela B. von Bonsdorff

**Affiliations:** 1 Gerontology Research Center and Faculty of Sport and Health Sciences, 541605 University of Jyväskylä, Jyvaskyla, Finland; 2 3812 Folkhälsan Research Center, Helsinki, Finland; 3 Clinicum, Faculty of Medicine, University of Helsinki, Helsinki, Finland; 4 Population Health Unit, Finnish Institute for Health and Welfare, Helsinki, Finland; 5 Department of General Practice and Primary Health Care, University of Helsinki and Helsinki University Hospital, Helsinki, Finland; 6 Department of General Practice and Primary Health Care, University of Helsinki, Helsinki, Finland; 7 Department of Obstetrics and Gynecology and Human Translational Research Programme, Yong Loo Lin School of Medicine, National University of Singapore, Singapore, Singapore; 8 Singapore Institute for Clinical Sciences (SICS), Agency for Science, Technology and Research (A*STAR), Singapore, Singapore

**Keywords:** social mobility, socioeconomic status, ageing, health-related quality of life

## Abstract

**Objectives:**

Changes in socioeconomic status (SES) during life may impact health in old age. We investigated whether social mobility and childhood and adulthood SES are associated with trajectories of health-related quality of life (HrQoL) over a 17-year period.

**Methods:**

We used data from the Helsinki Birth Cohort Study (*n* = 2003, 46% men, mean age 61.5 years). Social mobility was derived from childhood SES, obtained from healthcare records, and register-based adulthood SES.

**Results:**

Logistic regression models showed that lower adulthood SES was associated with lower physical HrQoL trajectories. Among men low (OR 3.95, *p* < .001), middle (OR 2.20, *p* = .006), and declining lifetime SES (OR 2.41, *p* = .001) were associated with lower physical HrQoL trajectories compared to men with high SES. Socioeconomic status was not associated with mental HrQoL trajectories.

**Discussion:**

Declining SES during life course may have negative health consequences, while improving SES is potentially as beneficial as high SES to later-life health among men.

## Introduction

Physical and mental functioning are key health-related factors when defining older people’s health-related quality of life (HrQoL). Previous studies strongly suggest that physical functioning declines with older age (Han et al., 2013; Kaplan et al., 1993; von Bonsdorff et al., 2019), while mental functioning seems to remain relatively stable (Avis et al., 2018; Blanchflower & Oswald, 2008; Jokela et al., 2010; Otero-Rodriguez et al., 2011; Tran et al., 2019). Nevertheless, some people succeed in preserving high levels of functioning in old age, which is associated with lower mortality rate (St. John et al., 2018), and later institutionalization (Luppa et al., 2010; von Bonsdorff et al., 2006).

Health disparities have been associated with several indicators of socioeconomic status (SES). People with higher SES tend to have better health, potentially because of, for example, healthier lifestyles and better access to healthcare (Blomgren & Virta, 2020; Kivimäki et al., 2020). Differences in early-life SES appear to have long-lasting effects on health (Hayward & Gorman, 2004). However, social mobility, (i.e. a positive or a negative change in SES) may play a role in defining health in later life (Cohen et al., 2010; Hallqvist et al., 2004; Luo & Waite, 2005). According to the ‘social mobility model’, the effect of childhood socioeconomic disadvantage on health can be reduced by upward social mobility in adulthood and vice versa, and a decline from higher SES to lower SES levels may lead to poorer health (Cohen et al., 2010; Hallqvist et al., 2004; Luo & Waite, 2005).

Previous studies have found that SES in childhood and adulthood are associated with the physical and mental dimensions of HRQoL (Hemingway et al., 1997; M. Laaksonen, Silventoinen, et al., 2007; Luo & Waite, 2005; M. B. von Bonsdorff, Kokko, et al., 2019). However, the longitudinal changes of HrQoL in old age appear to be somewhat unclear (N. A. Ross et al., 2012; Szabó et al., 2021). Previous studies of HrQoL propose that men may sustain better HRQoL than women (Hajian-Tilaki et al., 2017; Orfila et al., 2006) and the influence of socioeconomic conditions on health and quality of life may differ between sexes (Angelini et al., 2019; Landös et al., 2019; Luo & Waite, 2005; N. A. Ross et al., 2012). Despite prior investigations showing that lower early-life SES is linked to adverse changes in physical functioning in old age (Cheval et al., 2019; Haas, 2008; Kingston et al., 2015; Landös et al., 2019), only few studies have studied social mobility and trajectories of HrQoL in old age. Otero-Rodriguez et al. (2011) found that an improvement in social status from childhood to adulthood was associated with an improvement in HrQoL in old age during a 2-year follow-up. However, there is only sparse information on social mobility and the long-term trajectories of HrQoL in old age.

While many studies use self-reported retrospective data to define childhood SES, we were able to use objective register-based SES data from both childhood and adulthood. Combined with a considerably long follow-up time of 17 years and separate HrQoL trajectories for women and men, the current study expands earlier research and provides more information on the change in HrQoL in older age and how objective life course SES is associated with these trajectories. We aimed to investigate whether social mobility from childhood to adulthood is associated with trajectory classes of physical and mental components of HrQoL over a 17-year period in a sample of Finnish older men and women. In addition, we examined the association of childhood SES and adulthood SES individually with HrQoL trajectory classes. We assumed that lower SES throughout life and/or declining SES from childhood to adulthood is associated with a higher likelihood of belonging in lower HrQoL trajectory classes in older age compared to those with high SES, and that higher SES throughout life and/or improving SES from childhood to adulthood would be associated with higher likelihood of belonging in higher HrQoL trajectory classes throughout life.

## Methods

This study used information from The Helsinki Birth Cohort Study, a longitudinal study of 13,345 individuals born between 1934 and 1944 at the Helsinki University Central Hospital or Helsinki City Maternity Hospital in Helsinki, Finland. A random sample (*n* = 2902) of participants were invited to baseline clinical visit in 2001–2003 (*n* = 2003; mean age = 61.5 years), and two follow-up visits in 2011–2013 (invited *n* = 1404, participated *n* = 1094; mean age = 71.1 years) and 2017–2018 (invited *n* = 1174, participated *n* = 815; mean age = 75.9 years). A detailed description of the study population has been reported elsewhere (Eriksson et al., 2001; Haapanen et al., 2022). The participants provided a written informed consent. The study was approved by the Coordinating Ethics Committee of the Hospital District of Helsinki and Uusimaa and the Ethics Committee of National Public Health Institute, Helsinki.

### Main Variables

*Health-Related Quality of Life was* assessed in 2001–04, 2011–13, and 2017–18 using the validated Finnish version of the SF 36-Item Health Survey 1.0 (Aalto et al., 1999; Ware & Sherbourne, 1992). The 36 items measuring health and functioning were divided into two component scores. The physical component summary (PCS) included four domains: physical functioning (10 items), role limitations caused by physical health problems (4 items), bodily pain (2 items), and general health (5 items). The mental component summary (MCS) included four domains: role limitations caused by emotional problems (3 items), vitality (4 items), mental health (5 items), and social functioning (2 items). The domains were standardized using the means and standard deviations of the US reference population (1990) (Ware et al., 1993) and weighted using factor score coefficients from the same reference population. Physical (PCS) and mental component summary scores (MCS) were calculated from the standardized domains. Both scores were then rescaled to range between 0 and 100, with 100 representing the highest level of functioning or well-being. Latent class mixture models were used to extract classes of trajectories of PCS and MCS (see Ikonen et al., 2023), and the trajectory classifications were used as nominal variables in the main analyses below. Herein in this manuscript, we refer to these summary scores as physical component or PCS and mental component or MCS.

*Childhood SES* was composed of paternal occupation and household crowding in childhood. Father’s highest occupation was extracted from birth records, child welfare clinic records, and school healthcare records, and grouped as (1). Labourers, (2). Low officials (lower-level employees with administrative and clerical occupations), and (3). High officials (upper-level employees with administrative, managerial, professional, and related occupations). The classification was based on the classification used by the Central Statistical Office of Finland (1989). Data on household size and number of rooms was obtained from child welfare clinic records. Household crowding was calculated by dividing household size by the number of rooms.

*Adulthood SES* was composed of the highest education, highest occupational status during adulthood (i.e. social class), standardized household taxable income in 1990, and household crowding in 1990. Data were obtained at 5-year intervals between 1970 and 2000 from Statistics Finland. Educational attainment was coded as (1). Basic education or less or unknown, (2). Upper secondary, (3). Lower tertiary, and (4). Upper tertiary. The highest occupational status was coded as (1). Labourers, (2). Self-employed, (3). Low officials, and (4). High officials. For income, we used a square root equivalence scale to assign a value in proportion to each household’s needs (A. B. Atkinson et al., 1995). Household crowding in 1990 was calculated by dividing household size by the number of rooms.

Variables ‘childhood SES’ and ‘adulthood SES’ were derived from two separate principal component analyses based on polychoric correlation matrices. The PCA is a fairly common technique to construct SES variables (Vyas & Kumaranayake, 2006), and it was used as a tool for data compression. These variables were the scores for the first component and explained 69% (‘childhood SES’) and 47% (‘adulthood SES’) of the variance, respectively. The component scores of childhood and adulthood SES were divided into tertiles and classified as low SES, middle SES, and high SES. The classified childhood and adulthood SES variables were combined into ‘*social mobility’* variable with five categories: high SES (17%, *n* = 314, the reference category, including high childhood and adulthood SES), middle SES (12%, *n* = 208, including middle childhood and adulthood SES), low SES (12%, *n* = 225, including low childhood and adulthood SES), declining SES (31%, *n* = 559, including high childhood SES to middle or low adulthood SES and middle childhood SES to low adulthood SES), and improving SES (28%, *n* = 503, including low childhood SES to middle or high adulthood SES and middle childhood SES to high adulthood SES). Relationships between SES variables are shown in Supplementary Tables 5–11.

### Covariates

Chronic diseases were self-reported physician-diagnosed diseases or symptoms including hypertension, high cholesterol, diabetes, myocardial infarction, angina pectoris, congestive heart failure, claudication, osteoporosis, stroke, cancer, tuberculosis, depression, asthma, and emphysema. The number of chronic diseases was divided into three categories: no diseases, one chronic disease, and two or more chronic diseases. Smoking (1. Smoking 2. Not smoking) and alcohol consumption (1. Weekly, 2. Monthly or less, and 3. Not at all), were self-reported and used as descriptive information.

### Statistical Methods

PCS and MCS trajectory classes were calculated by using latent growth mixture models (LGMM) as part of a previous study (Ikonen et al., 2023). The models were fitted to the SF-36 physical and mental component summary scores from the three clinical examinations in 2001–2004, 2011–2013, and 2017–2018. The summary component scores were divided by 10 for the LGMM analysis. LGMM was used to find latent classes with similar physical and mental component trajectory classes. We used a growth model with time indexed through individually different observation times matching to the participants’ chronological age. Each latent class had its own growth parameters, intercept, and slope, and the average trajectory was based on the means of these parameters. The Bayesian Information Criterion (BIC) was used to observe the optimal number of latent classes. The analyses were run separately for men and women using Mplus version 7 (1998–2015). A detailed description of forming the physical and mental component trajectory classes has been reported elsewhere (Ikonen et al., 2023). Briefly, the trajectory classes were named based on their starting level and their graphical slope. Classes representing better HrQoL were named ‘high’ and trajectory classes with lower HrQoL were named ‘intermediate’ due to their graphical location in the middle of the range of the scale (Ikonen et al., 2023). Trajectory classes with only a little or no average trajectory slope at all were named as ‘stable’ and classes with a declining slope as ‘declining’. Two physical component average trajectory classes were identified for men and women. The trajectory classes were named ‘intermediate declining’ (34.4% of men and 44.5% of women) and ‘high declining’ (65.6% of men and 55.5% of women). Two mental component trajectory classes were identified for men and women. The trajectory classes were named ‘Intermediate stable’ (22.5% of men and 34.1% of women) and ‘High stable’ (77.5% of men and 65.9% of women). Hereafter in this manuscript, we refer to these trajectory classes as ‘high’ and ‘intermediate’.

Baseline characteristics of the participants were compared between trajectories using the Mann–Whitney *U*-test. For categorical variables, we used the chi-squared (*χ*^2^) test and Fisher’s exact test. Descriptive analyses were carried out with SPSS IBM version 28.0 (SPSS, Armonk, NY, IBM Corp). Associations between childhood and adulthood SES and PCS and MCS trajectory classes were modelled using logistic regression analyses, adjusting for birth year in Model 1. Model 2 for childhood SES was adjusted for birth year and adulthood SES. Model 2 for adulthood SES was adjusted for birth year and childhood SES. Associations between social mobility and MCS and PCS trajectory classes were analyzed using multinomial logistic regression. High PCS trajectory class and high MCS trajectory class were used as reference categories for the outcome variables, and the analyses were adjusted for birth year. The analyses were carried out with Stata (MP17.0). In addition, we carried out an additional sensitivity analysis for social mobility using only father’s occupation and adulthood occupation as indicators of childhood and adulthood SES, respectively (Supplementary Tables 3 and 4). All tests were performed two-tailed, and the hypothesis test result was called statistically significant for *p* < .05.

## Results

### Baseline Characteristics

Baseline characteristics among men and women according to the PCS trajectory classes are shown in Table 1. Among men, the mean PCS score in the intermediate trajectory class declined from 41.2 (SD ± 8.9) to 38.4 (SD ± 9.3) over the 17-year follow-up, and in the high trajectory class from 53.2 (SD ± 3.5) to 50.7 (SD ± 4.8). A larger percentage of men in the intermediate trajectory class had consistently low or declining SES during their lives than men in the high trajectory class. In addition, men in the intermediate trajectory class were more likely to have more than one chronic disease, and more likely to smoke but less likely to consume alcohol weekly relative to the high trajectory class. Regarding the adulthood characteristics, men in the intermediate trajectory class were more frequently less educated and had a lower social class and lower household income in adulthood (Table 1) than men in the high trajectory class. Differences were also found in childhood characteristics. As shown in Table 1, men in the intermediate trajectory class had slightly more people living per room in their childhood home and their father’s occupation was more likely to be in the labourers and less likely in the high official category. Among women, the mean PCS score in the intermediate PCS trajectory class declined from 39.7 (SD ± 9.3) to 35.5 (SD ± 8.7) over the 17-year follow-up, and in the high trajectory class from 52.8 (SD ± 3.8) to 50.6 (SD ± 4.9). Women in the intermediate trajectory class were on average slightly older, used less alcohol, and had more chronic diseases. In adulthood, they were slightly less educated and had a lower social class as well as lower household income in adulthood compared to the high trajectory (Table 1).

**Table 1.** Participants’ Demographic and Socioeconomic Characteristics by PCS Trajectory Classes.

	Physical Component (PCS) Trajectory Classes					
	Men			Women		
	Intermediate Declining (*n* = 317^a^)	High Declining (*n* = 604^a^)	*p*-Value	Intermediate Declining (*n* = 476^a^)	High Declining (*n* = 594^a^)	*p-*Value
Age, mean *(SD)*	61.7 (2.9)	61.5 (2.8)	0.495	62.0 (3.2)	61.2 (3.0)	<0.001
PCS in 2001–2003, *mean (SD)*	41.2 (8.9)	53.2 (3.5)	<0.001	39.7 (9.3)	52.8 (3.8)	<0.001
PCS in 2011–2013, *mean (SD)*	40.6 (9.7)	52.3 (3.9)	<0.001	39.0 (9.1)	51.8 (4.4)	<0.001
PCS in 2017–2018, *mean (SD)*	38.4 (9.3)	50.7 (4.8)	<0.001	35.5 (8.7)	50.6 (4.9)	<0.001
MCS in 2001–2003, *mean (SD)*	53.7 (10.2)	55.7 (6.8)	0.131	52.6 (10.5)	53.4 (8.9)	0.826
MCS in 2011–2013, *mean (SD)*	54.0 (9.8)	56.5 (6.1)	0.069	52.5 (11.0)	54.4 (7.7)	0.256
MCS in 2017–2018, *mean (SD)*	51.5 (11.1)	56.5 (6.7)	<0.001	51.8 (10.9)	54.2 (8.7)	0.016
Social mobility *n* (*%*)			<0.001			
High	34 (12)	115 (21)		72 (16)	93 (17)	0.159
Improving	54 (19)	177 (32)		111 (25)	161 (30)	
Middle	37 (13)	57 (10)		51 (12)	63 (12)	
Declining	107 (37)	151 (28)		138 (32)	163 (31)	
Low	56 (19)	48 (9)		66 (15)	55 (10)	
Married or cohabiting *n (%)*	257 (83)	527 (88)	0.055	305 (65)	414 (71)	0.054
Chronic diseases *n (%)*			<0.001			<0.001
No chronic diseases	63 (20)	248 (41)		104 (22)	260 (44)	
1 chronic disease	99 (31)	212 (35)		133 (28)	201 (34)	
2 or more chronic diseases	154 (49)	143 (24)		238 (50)	131 (22)	
Smoking *n (%)*	107 (34)	144 (24)	0.002	102 (22)	119 (20)	0.648
Alcohol use *n (%)*			0.012			0.010
3–7 times/week	55 (17)	153 (26)		54 (11)	65 (11)	
Weekly/monthly	198 (63)	357 (59)		244 (52)	357 (60)	
Rarely/not at all	62 (20)	91 (15)		174 (37)	169 (29)	
Education *n (%)*			<0.001			0.004
Basic or less/unknown	120 (38)	153 (25)		213 (45)	223 (37)	
Upper secondary	96 (30)	129 (22)		132 (28)	152 (26)	
Lower tertiary	77 (24)	195 (32)		100 (21)	156 (26)	
Upper tertiary	24 (8)	127 (21)		29 (6)	63 (11)	
Social class *n (%)*			<0.001			0.016
High official	45 (14)	137 (23)		41 (9)	62 (11)	
Low official	68 (22)	185 (30)		248 (52)	352 (59)	
Self-employed	30 (9)	65 (11)		42 (9)	49 (8)	
Labourer	174 (55)	217 (36)		144 (30)	131 (22)	
Household crowding 1990 *mean (SD)*	0.96 (0.51)	0.91 (0.46)	0.273	0.79 (0.34)	0.84 (0.39)	0.104
Annual income 1990 (€)* *mean (SD)*	45 859 (18 889)	53 676 (23 165)	<0.001	45 838 (23 615)	49 879 (24 426)	0.003
Fathers occupation *n (%)*			0.013			0.749
High official	46 (14)	131 (22)		69 (14)	95 (16)	
Low official	72 (23)	145 (24)		104 (22)	131 (22)	
Labourer	198 (63)	323 (54)		301 (64)	363 (62)	
Childhood household crowding *mean (SD)*	2.6 (1.2)	2.5 (1.2)	0.027	2.6 (1.3)	2.5 (1.2)	0.111

*Note.* Data are presented as the *mean* or *n* (*%*) at baseline if not stated otherwise. PCS, physical component summary score; MCS, mental component summary score. Mann–Whitney’s *U*-test for continuous variables due to non-normality, the chi-squared (*χ*^2^) test for categorical variables, and Fisher’s exact test for dichotomous categorical variables.

^a^Max. available *n*, *Household annual income per person equivalent to 2020 eur.

Baseline characteristics for men and women according to the MCS trajectory classes are shown in Table 2. Among men, the mean MCS scores stayed stable over the 17-year follow-up, with only a minor change from 44.6 (SD ± 10.3) to 45.0 (SD ± 10.9) in the intermediate trajectory class and from 58.0 (SD ± 3.6) to 58.1 (SD ± 4.3) in the high trajectory class. Compared to the high trajectory class, men in the intermediate trajectory class had a lower average household income in adulthood and fewer were married (Table 2). They had more often chronic diseases and more of them smoked compared to the high trajectory class. As seen in Table 2, the mean PCS score was also lower among men in the intermediate trajectory class. Among women, the mean MCS scores remained stable over the 17-year follow-up, with only a slight change from 44.1 (±10.9) to 45.3 (±10.7) in the intermediate trajectory class and from 57.7 (±4.2) to 58.0 (±4.9) in the high trajectory class. Women in the intermediate trajectory class were on average slightly older and had more often chronic diseases and a slightly lower PCS (Table 2).

**Table 2.** Participants’ Demographic and Socioeconomic Characteristics by MCS Trajectory Classes.

	Mental component (MCS) trajectory classes					
	Men			Women		
	Intermediate stable (*n* = 207^a^)	High stable (*n* = 714^a^)	*p-*value	Intermediate stable (*n* = 365^a^)	High stable (*n* = 705^a^)	*p*-value
Age, mean (SD)	61.8 (2.7)	61.5 (2.8)	0.121	61.9 (3.1)	61.3 (3.0)	0.003
PCS in 2001–2003, mean (SD)	47.1 (8.7)	49.6 (7.5)	0.013	45.4 (10.0)	47.8 (9.0)	<0.001
PCS in 2011–2013, mean (SD)	44.4 (10.0)	49.8 (7.3)	<0.001	43.7 (9.6)	47.7 (8.8)	<0.001
PCS in 2017–2018, mean (SD)	43.1 (10.0)	48.4 (7.7)	<0.001	41.9 (10.6)	45.6 (9.6)	<0.001
MCS in 2001–2003, mean (SD)	44.6 (10.3)	58.0 (3.8)	<0.001	44.1 (10.9)	57.6 (4.2)	<0.001
MCS in 2011–2013, mean (SD)	47.8 (10.4)	58.1 (4.0)	<0.001	46.6 (10.7)	58.0 (4.4)	<0.001
MCS in 2017–2018, mean (SD)	45.0 (10.9)	58.1 (4.3)	<0.001	45.3 (10.7)	58.0 (4.9)	<0.001
Social mobility *n* (%)			0.942			0.794
High	33 (18)	116 (18)		52 (16)	113 (17)	
Improving	51 (27)	180 (28)		90 (27)	182 (28)	
Middle	18 (10)	76 (12)		37 (11)	77 (12)	
Declining	59 (32)	199 (30)		110 (34)	191 (30)	
Low	25 (13)	79 (12)		39 (12)	82 (13)	
Married or cohabiting *n* (%)	159 (78)	625 (88)	<0.001	232 (65)	487 (70)	0.108
Chronic diseases *n* (%)			<0.001			<0.001
No chronic diseases	59 (29)	252 (35)		93 (26)	271 (39)	
1 chronic disease	53 (26)	258 (36)		113 (31)	221 (31)	
2 or more chronic diseases	93 (45)	204 (29)		157 (43)	212 (30)	
Smoking *n* (%)	68 (33)	183 (26)	0.040	77 (21)	144 (21)	0.811
Alcohol use *n* (%)			0.374			0.403
3–7 times/week	53 (26)	155 (22)		47 (13)	72 (10)	
Weekly/monthly	116 (57)	439 (62)		199 (55)	402 (57)	
Rarely/not at all	36 (17)	117 (16)		116 (32)	227 (33)	
Education *n* (%)			0.163			0.511
Basic or less/unknown	68 (33)	205 (29)		154 (42)	282 (40)	
Upper secondary	57 (27)	168 (24)		97 (27)	189 (27)	
Lower tertiary	49 (24)	223 (31)		89 (24)	167 (24)	
Upper tertiary	33 (16)	118 (16)		25 (7)	67 (9)	
Social class *n* (%)			0.772			0.631
High official	40 (19)	142 (20)		33 (9)	70 (10)	
Low official	52 (25)	201 (28)		210 (58)	390 (56)	
Self-employed	21 (10)	74 (10)		26 (7)	65 (9)	
Labourer	94 (46)	297 (42)		96 (26)	179 (25)	
Household crowding 1990 *mean* (SD)	0.96 (0.58)	0.91 (0.44)	0.885	0.81 (0.37)	0.82 (0.36)	0.264
Annual income 1990 (€)* mean (SD)	48 234 (21 888)	51 792 (22 104)	0.018	48 083 (27 737)	48 087 (22 097)	0.278
Fathers occupation *n* (%)			0.614			0.212
High official	43 (21)	134 (19)		54 (15)	110 (16)	
Low official	44 (21)	173 (24)		70 (19)	165 (23)	
Labourer	119 (58)	402 (57)		239 (66)	425 (61)	
Childhood household crowding *mean *(SD)	2.6 (1.2)	2.5 (1.2)	0.280	2.5 (1.3)	2.6 (1.2)	0.268

*Note.* Data are presented as the *mean* or *n* (*%*) at baseline if not stated otherwise. PCS, physical component summary score; MCS, mental component summary score. Mann–Whitney’s *U*-test for continuous variables due to non-normality, the chi-squared (*χ*^2^) test for categorical variables, and Fisher’s exact test for dichotomous categorical variables.

^a^Max. available *n*, *Household annual income per person equivalent to 2020 eur.

### Physical Component Trajectory Classes and SES

#### Childhood SES

As shown in Table 3, in Model 1 (adjusted for birth year), there was a significant difference in the odds of following the intermediate PCS trajectory between men in the middle childhood SES category compared to men in the high childhood SES category (OR 1.66, 95% CI 1.18–2.34). This association attenuated after controlling for adulthood SES in Model 2. Among women, there were no statistically significant differences in the odds of following the intermediate PCS trajectory between SES categories (Table 3).

**Table 3.** Odds Ratios for Intermediate PCS Trajectory Class Versus High PCS and for Intermediate MCS Trajectory Class Versus High MCS According to Childhood SES Categories (High SES as a Reference Category). Model 1 Adjusted for Birth Year, and Model 2 Adjusted for Birth Year and Adulthood SES.

Childhood SES	Model 1			Model 2		
	OR	95% CI	*p*-value	OR	95% CI	*p*-value
Intermediate PCS						
Men						
High	*Ref.*			*Ref.*		
Middle	1.66	1.18–2.34	0.004	1.26	0.87–1.81	0.225
Low	1.39	0.97–1.99	0.070	1.08	0.73–1.58	0.705
Women						
High	*Ref.*			*Ref.*		
Middle	0.83	0.61–1.13	0.247	0.75	0.54–1.02	0.068
Low	1.25	0.92–1.71	0.157	1.13	0.82–1.55	0.466
Intermediate MCS						
Men						
High	*Ref.*			*Ref.*		
Middle	1.01	0.69–1.50	0.946	0.91	0.60–138	0.657
Low	1.06	0.71–1.60	0.770	0.97	0.64–1.48	0.896
Women						
High	*Ref.*			*Ref.*		
Middle	0.97	0.70–1.34	0.848	0.96	0.69–1.33	0.785
Low	1.02	0.74–1.41	0.919	0.98	0.70–1.37	0.905

*Adulthood SES* was associated with PCS trajectory classes among men and women (Table 4). There was a significant difference in the odds of following the intermediate PCS trajectory among men in the low adulthood SES category and men in the middle adulthood SES category compared to men in the high adulthood SES category (OR 3.01, 95% CI 2.12–4.28; OR 1.66, 95% CI 1.15–2.37, respectively). Adjusting for birth year and childhood SES (Model 2, Table 4) led to minor changes (Low SES OR 3.17, 95% CI 2.17–4.64; Middle SES OR 1.78, 95% CI 1.22–2.62). These relative odds ratio effect sizes indicate a small to medium effect (Olivier et al., 2017). Among women, there was a significant difference in the odds of following the intermediate PCS trajectory among women in the low adulthood SES category and women in the middle adulthood SES category compared to women in the high adulthood SES category (Low SES OR 1.45, 95% CI 1.07–1.96; Middle SES OR 1.46, 95% CI 1.08–1.98, respectively). The associations remained statistically significant after further adjustment for childhood SES in Model 2 (Low SES OR 1.49, 95% CI 1.08–2.05; Middle SES OR 1.60, 95% CI 1.16–2.20).

**Table 4.** Odds Ratios for Intermediate PCS Trajectory Class Versus High PCS Trajectory Class, and for Intermediate MCS Trajectory Class Versus High MCS Trajectory Class According to Adulthood SES Categories (High SES as a Reference Category). Model 1 Adjusted for Birth Year, Model 2 Adjusted for Birth Year and Childhood SES.

Adulthood SES	Model 1			Model 2		
	OR	95% CI	*p–Value*	OR	95% CI	*p–Value*
Intermediate PCS						
Men						
High	*Ref.*			*Ref.*		
Middle	1.66	1.15–2.37	0.006	1.78	1.22–2.62	0.003
Low	3.01	2.12–4.28	<0.001	3.17	2.17–4.64	<0.001
Women						
High	*Ref.*			*Ref.*		
Middle	1.46	1.08–1.98	0.014	1.60	1.16–2.20	0.004
Low	1.45	1.07–1.96	0.016	1.49	1.08–2.05	0.016
Intermediate MCS						
Men						
High	*Ref.*			*Ref.*		
Middle	1.03	0.69–1.53	0.882	0.99	0.66–1.50	0.974
Low	1.41	0.97–2.07	0.075	1.35	0.89–2.05	0.157
Women						
High	*Ref.*			*Ref.*		
Middle	1.05	0.77–1.43	0.774	1.18	0.85–1.65	0.327
Low	1.03	0.75–1.41	0.854	1.08	0.77–1.52	0.653

**Figure 1.**
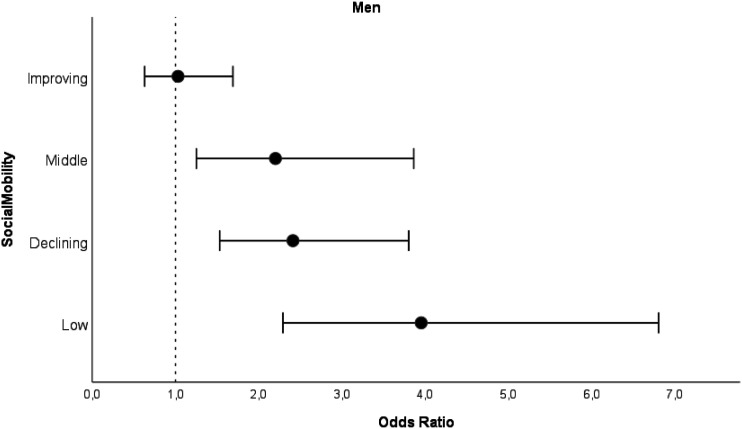
Odds ratios for intermediate PCS trajectory class with 95% confidence intervals for men by social mobility categories compared to high PCS trajectory class. *Note*. Analyzed with logistic regression analysis. Model adjusted for birth year. High SES as a reference category.

**Figure 2.**
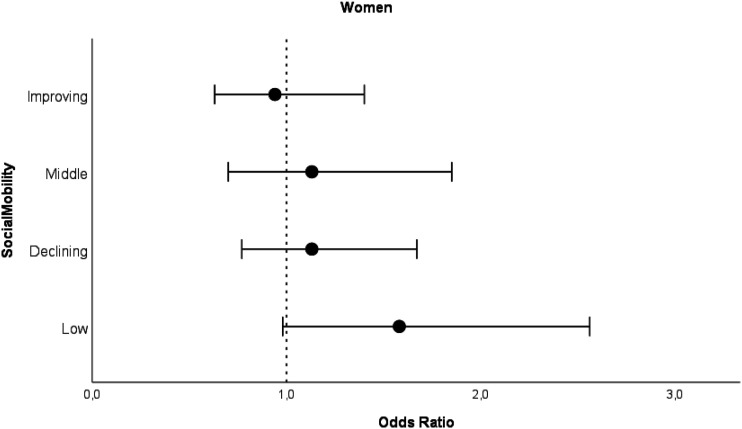
Odds ratios for intermediate PCS trajectory class with 95% confidence intervals for women by social mobility categories compared to high PCS trajectory class*. Note.* Analyzed with logistic regression analysis. Model adjusted for birth year. High SES as a reference category.

*Social mobility* was associated with PCS trajectory classes among men, but not women (Figure 1 and Supplementary Table 1). Men with low (OR 3.95, 95% CI 2.29–6.80), middle (OR 2.20, 95%CI 1.25–3.86), or declining SES (OR 2.41, 95% CI 1.53–3.80) throughout life had a significant difference in the odds of being in the intermediate PCS trajectory class compared to men with high SES throughout life, with relative effect sizes indicating a medium effect (Olivier et al., 2017). Improving SES (OR 1.03 95% CI 0.63–1.69) was not associated with intermediate PCS trajectory classes among men. Among women, there was no significant difference in the odds of following the intermediate PCS trajectory between social mobility categories compared to women in the high SES category throughout life (Figure 2 and supplementary Table 1).

In the sensitivity analysis (Supplementary Table 3), the results were largely similar. An exception was improving SES among men. Men with improving SES had a significant difference in the odds (OR 2.18, C.I 95% 1.06–4.48) of following the intermediate PCS trajectory compared to men with high SES, although the confidence intervals were wide.

### Mental Component Trajectory Classes and SES

**Figure 3.**
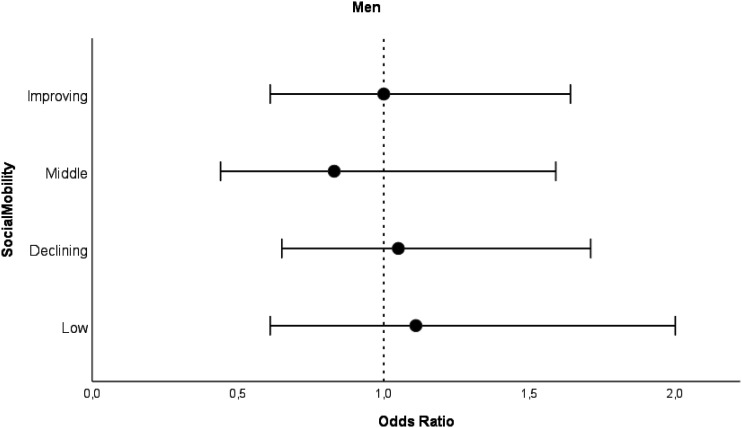
Odds ratios for intermediate MCS trajectory class with 95% confidence intervals for men by social mobility categories compared to high MCS trajectory class. *Note*. Analyzed with logistic regression analysis. Model adjusted for birth year. High SES as a reference category.

**Figure 4.**
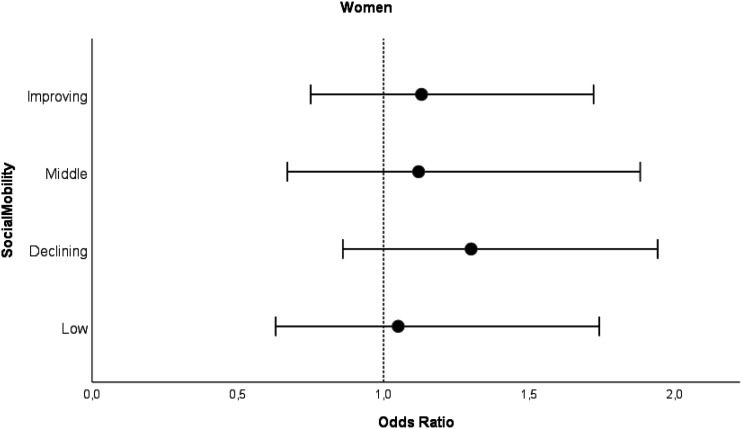
Odds ratios for intermediate MCS trajectory class with 95% confidence intervals for women by social mobility categories compared to high MCS trajectory class. *Note.* Analyzed with logistic regression analysis. Model adjusted for birth year. High SES as a reference category.

When we analyzed childhood and adulthood SES separately, we found no associations with MCS trajectory classes (Table 3 and Table 4). Social mobility was not associated with MCS trajectory classes in either sex (Figures 3 and 4, and Supplementary Table 2). In the sensitivity analysis (Supplementary Table 4), the results were similar and no associations were observed.

## Discussion

We found that downward social mobility from childhood to adulthood, as well as low and middle SES across the life course were statistically significantly associated with continuously lower PCS trajectory class in old age among men over a 17-year follow-up. Among women, we found no statistically significant associations between social mobility and PCS trajectory classes. MCS trajectory classes were not associated with social mobility or SES in either men or women.

To the best of our knowledge, this study is one of the few that has studied the association between life course social mobility derived from national registers and trajectories of HrQoL in older age with a notably long follow-up time. Our findings support the social mobility model to some extent and are in line with previous studies indicating that changes across life course in SES are associated with changes in health and quality of life in old age (Letelier et al., 2021; Otero-Rodriguez et al., 2011). Notably, participants with improving SES in our study did not show a clear difference in PCS trajectory compared to participants with high SES. In other words, men with improving SES had physical health similar to men with consistently high SES, possibly indicating that an improvement in SES may be as beneficial to health as having high SES throughout life. However, this interpretation needs to be approached with some caution, as the sensitivity analysis suggested a difference, although with wide confidence intervals. Nevertheless, the findings suggest that by preventing decline in SES or perhaps by improving one’s SES during life course, it may be possible, to a moderate degree, to prevent low health and functioning later in life.

Individuals with a higher SES often enjoy better access to healthcare (Blomgren & Virta, 2020; McMaughan et al., 2020), even in the Nordic welfare system, in which healthcare is available to everyone regardless of financial condition. In addition, higher SES is linked to a healthier lifestyle (Hiscock et al., 2012; Øvrum, 2011; J. Wang & Geng, 2019), less experiences of financial strain in old age (Harber-Aschan et al., 2020), and proper nutrition (Atkins et al., 2015), which all contribute to improved health outcomes in old age. People with high SES tend to have better life circumstances, more social support, and greater social activity (B. Wang et al., 2023), which can act as effective stress control mechanisms, and thus promote health in old age. Individuals facing financial difficulties and limited social resources may be more susceptible to chronic stress, which can later impact physical health (Brunner & Marmot, 2006). Consequently, it might be possible to maintain or promote better quality of life of older people through policies and economic strategies that aim for equality in education and employment, for example. Such policies may benefit the health and quality of life of those in the lowest SES (C. E. Ross & Mirowsky, 2010). This, in turn, may promote health equity and further diminish health disparities.

Based on a previous review (Hays & Morales, 2001; Samsa et al., 1999), a 3–5-point difference in the SF-36 scores may indicate a small clinically important effect. Therefore, the differences in the PCS scores between the trajectory classes can be considered clinically meaningful, as the difference in the average PCS scores between high and intermediate trajectory classes were 12–15 points. The PCS scores differed also between the social mobility groups at different follow-ups, with a maximum difference of 5 points (Supplementary Table 6).

We did not find an association between childhood SES and PCS trajectories in old age when adulthood SES was taken into account. The finding is in line with some previous studies on physical health (Cheval et al., 2019; M. Laaksonen, Martikainen, et al., 2007) while most earlier studies have contrasting results (Haas, 2008; Otero-Rodriguez et al., 2011; Zimmer et al., 2016). The analysis suggested that the effect of childhood SES may be mediated by adulthood SES.

The associations between social mobility and HrQoL were absent among women in our study. Women in our study smoked less and used less alcohol at baseline than men, which may indicate that they have adopted more healthy habits and therefore the impact of SES and social mobility may not be visible. In addition, health disparities based on social factors may be more clear among men than women (Elo, 2009; Martikainen et al., 2004). Furthermore, the employment and education status of women in different cultures and societies needs to be considered. In earlier decades, the husband usually had a higher SES than the wife, which significantly influenced the family’s wealth and lifestyle. In 1970, about 57% of working aged women in Finland were in the labour force, in comparison to 79% of working aged men (Statistics Finland, 1993). By 1980, the labour force participation rate for women was 61% and 74% for men. Factors like these may make it challenging to determine women’s individual SES based on their education and employment. In addition, as Steiber (2019) suggests, the influence of social mobility on health may diminish with age, and perhaps this partly explains our results for women.

The trajectory classes of MCS in our study remained relatively stable during the 17-year follow-up, in accordance with previous studies (Blanchflower & Oswald, 2008; Jokela et al., 2010; Otero-Rodriguez et al., 2011). Contrary to our hypotheses, we did not find any statistically significant associations between SES or social mobility and trajectories of MCS in old age. This finding is in contrast with previous studies on SES and mental health (Angelini et al., 2019; Luo & Waite, 2005; Otero-Rodriguez et al., 2011; Pinquart & Sörensen, 2000), although some similar findings have been reported, for example, by J. Wang & Geng (2019). In addition, some studies have pondered that the association between SES and mental health is strongly affected by how SES is measured (E. Laaksonen, Martikainen, et al., 2007; M. Laaksonen, Silventoinen, et al., 2007; Lahelma et al., 2006), which may play a role in our findings. E. Laaksonen, Martikainen, et al. (2007) and Lahelma et al. (2006) reported that economic indicators may be more important to mental health than conventional SES indicators. In addition, the observed trajectories of MCS in our study were less stable and there was more interindividual variability between the observed trajectories (Ikonen et al., 2023, supplementary Figure 3) than in the observed PCS trajectories. This may play a role in the lack of association between MCS and SES. Finally, there may be psychological characteristics or coping mechanisms that are related to SES, which we were unable to observe in this study.

The main strengths of this study are the longitudinal study design and data from a unique birth cohort. In addition to self-reported survey data, we included several objective SES indicators derived from national registers. The SF-36 is a widely validated and commonly used measure (Ware & Gandek, 1998), which has been previously used in longitudinal studies (Hemingway et al., 1997; Mishra et al., 2014; St. John et al., 2018), making our results comparable to other studies. Regarding the trajectories, we believe that separate trajectories for men and women are another strength of our study. Health disparities between sexes are more common in older ages (e.g. life expectancy and morbidity), which needs to be considered when creating trajectories of health and functioning in older age.

This study has also some limitations. As often happens in studies focusing on the ageing population, people with functional decline or severe diseases may choose not to participate in the first place or drop out later. Thus, some of the lower rates or deepest declines in PCS or MCS may not be visible in our study. In addition, participants in our study were on average doing well financially, indicating that the individuals with very low socioeconomic position may not be included in our study. Second, our SES variables did not cover the whole lifespan of participants, such as young adulthood. Furthermore, to examine changes in SES from childhood to adulthood, we chose to derive SES variables from categorized principal components. Such an approach reduces variation in the data and may not entirely reflect the socioeconomic levels in the population. Further, partly different variables were used as the basis for SES principal components in childhood and adulthood because of the limited availability of SES indicators. Hence, caution should be taken when interpreting the results on social mobility, although the results of the sensitivity analysis were very similar. Finally, trajectories compress a lot of information and thereby some variation in the data is inevitably being missed. Future studies should focus on longitudinal follow-up studies in diverse populations.

## Conclusions

The present study aimed to clarify the association between social mobility and the change in HrQoL across older age. Our results support the social mobility model suggesting that a declining SES from childhood to adulthood may have negative health consequences, and that an improving SES may possibly be as beneficial to later-life health as maintaining a high SES throughout life. The present study suggests that in order to reduce disparities in health across older age, we need to reduce SES inequalities earlier in life.

## Supplemental Material

Supplemental Material - Social Mobility and Health-Related Quality of Life Trajectory Classes Among Older Women and Men Supplemental Material for Social Mobility and Health-Related Quality of Life Trajectory Classes Among Older Women and Men by Anna-Maria Lahti, Tuija M. Mikkola, Niko S. Wasenius, Timo Törmäkangas, Jenni N. Ikonen, Sini Siltanen, Johan G. Eriksson, and Mikaela B. von Bonsdorff
