# Supplementary material for: Social Mobility and Health-Related Quality of Life Trajectory Classes Among Older Women and Men
Source: J Aging Health. 2024 Apr 1;37(3-4):220–32. doi: 10.1177/08982643241242513 (PMC11829508; doi:10.1177/08982643241242513)
Supplement: Supplemental Material - Social Mobility and Health-Related Quality of Life Trajectory Classes Among Older Women and Men [file sj-pdf-1-jah-10.1177_08982643241242513.pdf]

SUPPLEMENTAL MATERIAL

Supp. table 1. *Odds ratios for intermediate PCS trajectory class according to social mobility categories among men and women.*

Social mobility	N	OR	95% C.I.	*p*–value*
Men				
High	149	*Ref.*		
Improving	231	1.03	0.63–1.69	0.894
Middle	94	2.20	1.25–3.86	0.006
Declining	258	2.41	1.53–3.80	<0.001
Low	104	3.95	2.29– 6.80	<0.001
Women				
High	165	*Ref.*		
Improving	272	0.94	0.63–1.40	0.764
Middle	114	1.13	0.70–1.85	0.610
Declining	301	1.13	0.77–1.67	0.522
Low	121	1.58	0.98–2.56	0.060

Note. Adjusted for birth year. *p-value compared to stable high SES.

Supp. table 2. *Odds ratios for intermediate MCS trajectory class according to social mobility categories among men and women.*

Social mobility	N	OR	95% C.I.	*p*–value*
Men				
High	149	*Ref.*		
Improving	231	1.00	0.61–1.64	0.999
Middle	94	0.83	0.44–1.59	0.578
Declining	258	1.05	0.65–1.71	0.836
Low	104	1.11	0.61–2.00	0.729
Women				
High	165	*Ref.*		
Improving	272	1.13	0.75–1.72	0.561
Middle	114	1.12	0.67–1.88	0.657
Declining	301	1.30	0.86–1.94	0.211
Low	121	1.05	0.63–1.74	0.861

Note. Adjusted for birth year. *p-value compared to stable high SES.

Supp. table 3. *Odds ratios for intermediate PCS trajectory class according to social mobility categories consisted of father's occupation and occupation in adulthood among men and women.*

Social mobility (occupation)	N	OR	95% C.I.	*p*–value*
Men				
High	65	*Ref.*		
Improving	290	2.18	1.06–4.48	0.034
Middle	91	2.34	1.04–5.25	0.040
Declining	192	3.00	1.44–6.26	0.003
Low	277	4.92	2.41–10.04	<0.001
Women				
High	34	*Ref.*		
Improving	498	1.05	0.51– 2.15	0.890
Middle	149	1.09	0.51–2.35	0.826
Declining	183	1.26	0.59–2.67	0.552
Low	198	1.70	0.81–3.59	0.164

Note. Adjusted for birth year. *p-value compared to stable high SES.

Supp. table 4. *Odds ratios for intermediate MCS trajectory class according to social mobility categories consisted of father's occupation and occupation in adulthood among men and women.*

Social mobility (occupation)	N	OR	95% C.I.	*p*–value*
Men				
High	65	*Ref.*		
Improving	290	0.86	0.46–1.60	0.626
Middle	91	0.56	0.25–1.25	0.158
Declining	192	1.05	0.55–2.00	0.894
Low	277	0.95	0.51–1.78	0.879
Women				
High	34	*Ref.*		
Improving	498	0.79	0.39–1.60	0.518
Middle	149	0.63	0.30–1.36	0.243
Declining	183	0.66	0.31–1.39	0.268
Low	198	0.85	0.41–1.78	0.663

Note. Adjusted for birth year. *p-value compared to stable high SES.

Supp. table 5. *Descriptives of childhood SES characteristics in groups of categorized SES component score.*

	Childhood SES		
Characteristic	Low	Middle	High
Fathers occupation, *n (%)*			
Labourers	509 (89)	537 (85)	60 (9.4)
Low officials	56 (9.8)	74 (11.7)	296 (46.5)
High officials	7 (1.2)	21 (3.3)	281 (44.1)
Childhood household crowding, *mean (SD)*	3.97 (1.04)	2.16 (0.56)	1.63 (0.51)

Supp. table 6. *Descriptives of adulthood SES characteristics in groups of categorized SES component score.*

	Adulthood SES		
Characteristic	Low	Middle	High
Education, *n (%)*			
Basic or less/ unknown	433 (66.6)	239 (36.5)	24 (3.7)
Upper secondary	187 (28.8)	235 (35.9)	79 (12.1)
Lower tertiary	30 (4.6)	165(25.2)	326 (49.8)
Upper tertiary	0 (0)	15 (2.3)	225 (34.4)
Social class, *n (%)*			
Labourer	503 (77.4)	150 (22.9)	3 (0.5)
Self-employed	46 (7.1)	85(13)	52 (8)
Low official	100 (15.4)	402 (61.5)	335 (51.2)
High official	1 (0.2)	17 (2.6)	264 (40.4)
Household crowding 1990, *mean (SD)*	1.04 (0.54)	0.83 (0.34)	0.72 (0.26)
Annual income 1990 (€), *mean (SD)*	34,766 (12,854)	47,044 (14,482)	66,836 (26,984)

Supp. table 7. *Correlations of SES component scores and categorized SES variables.*

	Childhood SES component score
Adulthood SES component score	Pearson's r 0.233
	Childhood SES categorized
Adulthood SES categorized	Spearman's r 0.266

Supp. table 8. *Descriptives of childhood and adulthood SES characteristics and the SF-36 summary scores in groups of categorized SES component score.*

	Social mobility				
	Low	Declining	Middle	Improving	High
Fathers occupation, *n (%)*					
Labourer	202 (89.8)	259 (46.3)	171 (82.2)	439 (87.3)	21 (7.6)
Low official	21 (9.3)	183 (32.7)	28 (13.5)	51 (10.1)	129 (41.1)
High official	2 (0.9)	117 (20.9)	9 (4.3)	13(2.6)	164 (52.2)
Childhood household crowding, mean (SD)	4.01 (1.16)	1.88 (.55)	2.19 (.58)	3.36 (1.20)	1.57 (.50)
Education, *n (%)*					
Basic or less/ unknown	158 (70.2)	296 (53)	82 (39.4)	97 (19.3)	5 (1.6)
Upper secondary	60 (26.7)	174 (31.1)	76 (36.5)	123 (24.5)	28 (8.9)
Lower tertiary	7 (3.1)	82 (14.7)	46 (22.1)	197 (39.2)	150 (47.8)
Upper tertiary	0 (0)	7 (1.3)	4 (1.9)	86 (17.1)	131 (41.7)
Social class, *n (%)*					
Labourer	169 (75.1)	331 (59.2)	51 (24.5)	53 (10.5)	1 (0.3)
Self-employed	20 (8.9)	53 (9.5)	20 (9.6)	48 (9.5)	30 (9.6)
Low official	36 (16.0)	166 (29.7)	129 (62)	293 (58.3)	145 (46.2)
High official	0 (0)	9 (1.6)	8 (3.8)	109 (21.7)	138 (43.9)
Household crowding 1990, *mean (SD)*	1.04 (.46)	0.99 (0.54)	0.84 (.33)	0.74 (0.26)	0.72 (0.27)
Annual income 1990 (€), *mean (SD)*	35.771 (13.147)	38.012 (14.386)	47.061 (15.817)	59.729 (24.808)	66.061 (27.650)
The SF-36 scores, *mean (SD)*					
PCS 2001	44.59 (9.9)	47.26 (9.26)	48.39 (8.18)	49.11 (8.3)	49.30 (8.6)
PCS 2011	45.05 (9.89)	46.45 (9.8)	46.03 (9.59)	47.89 (8.55)	48.87 (7.95)
PCS 2017	42.57 (10.02)	46.30 (9.61)	43.42 (10.10)	46.11 (9.08)	45.96 (9.82)
MCS 2001	53.68 (8.69)	54.09 (9.37)	54.77 (8.27)	53.74 (9.02)	54.30 (8.91)
MCS 2011	53.86 (10.47)	54.28 (9.27)	54.92 (8.12)	55.10 (7.94)	55.01 (7.40)
MCS 2017	52.49 (9.75)	53.63 (10.40)	53.84 (9.15)	54.88 (8.51)	54.53 (8.05)

Supp. table 9a. *SES variable associations within-era (childhood and adulthood).*

CHILDHOOD				
	Father's highest occupational status			
	Labourer	Low offical	High official	
Childhood household crowding (*mean, CI*)	2.81 (2.75,2.88)	2.27 (2.16,2.38)	1.91 (1.78,2.04)	
ADULTHOOD				
	Highest occupational status			
	Labourer	Self-employed	Low official	High official
Highest education	(n)	(n)	(n)	(n)
Basic or less/ unknown	342	39	320	11
Upper secondary	267	46	189	12
Lower tertiary	58	65	292	116
Upper tertiary	4	37	57	147
Household income (*mean, CI*)	-.385 (-.456,-.314)	.013 (-.122,.148)	.080 (.017,.143)	.665 (.555,.774)
Household crowding *(mean, CI*)	.961 (.929,.993)	.847 (.787,.908)	.817 (.788,.845)	.802 (.754,.851)

Supp. table 9b. *Correlations of continuous SES variables in adulthood.*

	Highest education	Household income	Household crowding
Highest education	1		
Household income	Spearman *r* 0.285	1	
Household crowding	Spearman *r* -0.078	Pearson *r* -0.154	1

Supp. table 9c. *SES variable associations between-era (childhood and adulthood).*

ADULTHOOD VARIABLES	CHILDHOOD VARIABLES		
	Father's highest occupational status		
	Labourer (n)	Low official (n)	High official (n)
Education in adulthood			
Basic or less/ unknown	516	132	58
Upper secondary	344	100	67
Lower tertiary	245	151	131
Upper tertiary	88	70	87
Highest occupational status in adulthood			
Labourer	478	134	54
Self-employed	93	51	43
Low official	516	189	146
High official	105	79	100
Household income in adulthood (mean, CI)	-.088 (-.144,-.033)	.071 (-.025,.167)	.225 (.119,.331)
Household crowding in adulthood (mean, CI)	.878 (.855,.901)	.867 (.820,.913)	.817 (.777,.856)
		Childhood household crowding	
Highest occupational status in adulthood (mean, CI)			
Labourer		2.74 (2.63,2.84)	
Self-employed		2.56 (2.38,2.74)	
Low official		2.52 (2.43,2.60)	
High official		2.15 (2.02,2.27)	
	Pearson *r*	Spearman *r*	
Adulthood household income	-0.056		
Adulthood household crowding	0.035		
Adulthood highest education		-.212	

PCA ANALYSIS CHILDHOOD SES

Supp. table 10a. *Principal component analysis*

		Variance explained	
manuPrincipal component	Eigenvalue	Proportion	Cumulative proportion
1	1.37851	0.6893	0.6893
2	0.62149	0.3107	1.0000

Supp. table 10b. *Principal component loadings for first principal component.*

Variable	Principal component	Uniqueness
Father's occupation	-0.5108	0.7391
Crowding	0.5108	0.7391

PCA ANALYSIS ADULTHOOD SES

Supp. table 11a. *Polychoric correlation matrix for adulthood SES*

	Income	Social class	Education	Crowding
Income 1990	1			
Social class	.35766786	1		
Education	.31322104	.56275636	1	
Crowding 1990	-.15360074	-.16801024	-.09493108	1

Supp. table 11b. *Principal component analysis*

		Variance explained	
Principal component	Eigenvalue	Proportion	Cumulative proportion
1	1.89660	0.4741	0.4741
2	0.94939	0.2373	0.7115
3	0.72335	0.1808	0.8923
4	0.43066	0.1077	1.0000

Supp. table 11c. *Principal component loadings for first principal component*

Variable	Principal component	Uniqueness
Income 1990	0.6699	0.5512
Social class	0.8319	0.3080
Education	0.7937	0.3700
Crowding 1990	-0.3547	0.8742
